# classifieR a flexible interactive cloud-application for functional annotation of cancer transcriptomes

**DOI:** 10.1186/s12859-022-04641-x

**Published:** 2022-03-31

**Authors:** Gerard P. Quinn, Tamas Sessler, Baharak Ahmaderaghi, Shauna Lambe, Harper VanSteenhouse, Mark Lawler, Mark Wappett, Bruce Seligmann, Daniel B. Longley, Simon S. McDade

**Affiliations:** 1 Patrick G Johnston Centre for Cancer Research, Queen’s University Belfast, 97 Lisburn Road, Belfast, BT9 7AE Northern Ireland, UK; 2 Electronics, Electrical Engineering and Computer Science, Queen’s University Belfast, Belfast, UK; 3 BioClavis, Glasgow, UK; 4 BioSpyder Technologies, Carlsbad, CA USA

**Keywords:** Colorectal Shiny CMS CRIS Immune, Cancer Subtype, Functional Annotation, Gene expression, Shiny application

## Abstract

**Background:**

Transcriptionally informed predictions are increasingly important for sub-typing cancer patients, understanding underlying biology and to inform novel treatment strategies. For instance, colorectal cancers (CRCs) can be classified into four CRC consensus molecular subgroups (CMS) or five intrinsic (CRIS) sub-types that have prognostic and predictive value. Breast cancer (BRCA) has five PAM50 molecular subgroups with similar value, and the OncotypeDX test provides transcriptomic based clinically actionable treatment-risk stratification. However, assigning samples to these subtypes and other transcriptionally inferred predictions is time consuming and requires significant bioinformatics experience. There is no "universal" method of using data from diverse assay/sequencing platforms to provide subgroup classification using the established classifier sets of genes (CMS, CRIS, PAM50, OncotypeDX), nor one which in provides additional useful functional annotations such as cellular composition, single-sample Gene Set Enrichment Analysis, or prediction of transcription factor activity.

**Results:**

To address this bottleneck, we developed classifieR, an easy-to-use R-Shiny based web application that supports flexible rapid single sample annotation of transcriptional profiles derived from cancer patient samples form diverse platforms. We demonstrate the utility of the " classifieR" framework to applications focused on the analysis of transcriptional profiles from colorectal (classifieRc) and breast (classifieRb). Samples are annotated with disease relevant transcriptional subgroups (CMS/CRIS sub-types in classifieRc and PAM50/inferred OncotypeDX in classifieRb), estimation of cellular composition using MCP-counter and xCell, single-sample Gene Set Enrichment Analysis (ssGSEA) and transcription factor activity predictions with Discriminant Regulon Expression Analysis (DoRothEA).

**Conclusions:**

classifieR provides a framework which enables labs without access to a dedicated bioinformation can get information on the molecular makeup of their samples, providing an insight into patient prognosis, druggability and also as a tool for analysis and discovery. Applications are hosted online at https://generatr.qub.ac.uk/app/classifieRc and https://generatr.qub.ac.uk/app/classifieRb after signing up for an account on https://generatr.qub.ac.uk.

**Supplementary Information:**

The online version contains supplementary material available at 10.1186/s12859-022-04641-x.

## Background

Next generation sequencing (NGS) is leading the drive towards personalised precision cancer medicine. Single sample annotation of cancer patient samples, the process of annotating each sample individually with transcriptionally inferred sub-types and functional predictions are increasingly important to understand disease aetiology and underlying biological mechanisms. Within CRC the best-established sub-grouping methodology to date is the consensus molecular subgroups (CMS1-4) [1], which is signficantly influenced by stromal (CMS4) and immune cell (CMS1) infiltration. The complementary Colorectal Intrinsic Subgrouping (CRIS) [2] focuses on cancer-cell intrinsic transcriptional features and overcomes the confounding effects of regional sampling and tumour heterogeneity [3]. Work from our group and others suggest that the CMS/CRIS classifiers have prognostic [4, 5] and predictive utility [6]. Moreover, these studies and recent progress in understanding the impact of cellular composition such as immune infiltration in predicting patient outcomes [6] highlight the utility of further annotating CRC transcriptional profiles with functional predictions using tools such as; (i) Microenvironment Cell Populations-counter (MCP-Counter) [7] and (ii) xCell for cellular composition [8]; (iii) single sample geneset enrichment analysis (ssGSEA) [9] for pathways and (iv) Discriminant Regulon Expression Analysis (DoRothEA) for transcription factor activity predictions [10]. Similarly, breast cancer (BRCA) patients can be stratified into 4 molecularly defined PAM50 subgroups with similar predictive and prognostic utility to the CRC classifications [11] or risk stratified through inference of clinical signatures such as OncotypeDX [12]. While the R-based tools required for such annotation of transcriptional profiles are freely available, this process can be time-consuming, error-prone and requires computational skills which are not available in all labs or where available is time-consuming to reproduce. Importantly, no single streamlined platform currently exists expedite these processes reproducibly.

**Fig. 1:**
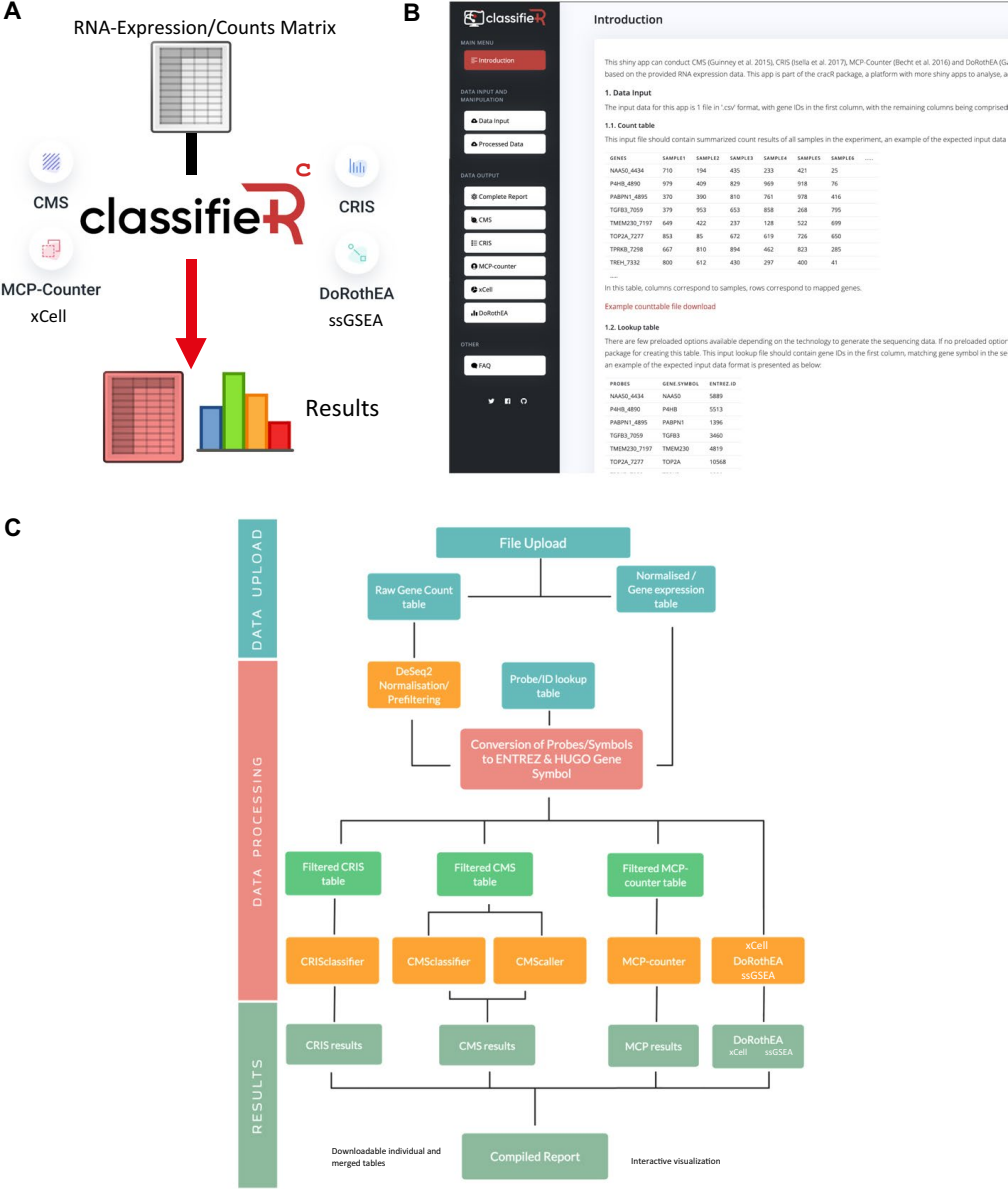
Processes in the backend of the classifieR^c^ shiny application. **a** Visual abstract of classifieR application, **b** Screenshot of the GUI of classifieR^c^ data input page. **c** Schematic overview of classifieR^C^ architecture and sub-functions

To address these challenges, we utilised Shiny, a web-based wrapper for R-based analyses to develop the user friendly classifieR framework. The core classifieR app functionalities enable efficient customisable analysis and annotation of gene expression data from a range of R-based packages, as exemplified by the CRC-specific application classifieR^C^, which in its current version (1.0) enables intuitive click-of-a-button annotation of CRC transcriptomic datasets. This analysis includes transcriptomic subtyping through CRIS and CMS and its derivative CMScaller sub-types (CMS adapted for PDX models and cell lines) to help summarise patient biology. An assessment of cellular content using MCP-counter and xCell which can be used to detect which cells are present in the tumour microenvironment (TME). Prediction of transcription factor activity scores with DoRothEA, and providing Single Sample Geneset Enrichment (ssGSEA) analysis (Fig. 1) which can provide an indication as to what oathways may be active within the tumour and the TME, as well as changes in TF activity which may underlie this observed biology.

## Implementation

classifieR was developed in a R environment [13] using Shiny (Shiny,RRID:SCR_001626) [14] that allows the R code to run within a HTML and JavaScript framework. The classifieR applications have purposefully been designed with a modern user-friendly graphical user interface (GUI) with detailed information on each of the tools as well as clear instructions how to perform, refine and interpret each of the provided classification/annotation tools (Fig. 1). The user can upload either a log2 normalised gene expression matrix, a DeSeq2 normalised expression matrix or raw gene counts from a variety of platforms including standard RNA sequencing, Affymetrix arrays and the TempO-Seq platform [15]. classifieR also enables users to upload and normalize their own RNA sequencing raw count data through DESeq2 (DESeq, RRID:SCR_000154) if not already completed prior to use of the tool. Therefore, classifieR can take input from any transcriptome sequencing or assay platform. The user then selects which of the R packages that they wish to execute (CMSclassifier, CRISclassifier (classifieRc), PAM50, OncotypeDX (classifieRb) DoRoTheA, xCell and MCP-counter); these have been modified internally to improve speed of functionality that they wish to execute (Fig. 1b) when compared to the original packages, saving time for the end user. Results are then presented as a summary report, interactive summary plots and results in a single table that can be downloaded as a.csv file. Results from each R-package can be further interrogated within individual tabs, which contains more detailed tabular information, including reactive graphical representations of the results, all of which can also be downloaded in figure ready formats.

**Fig. 2:**
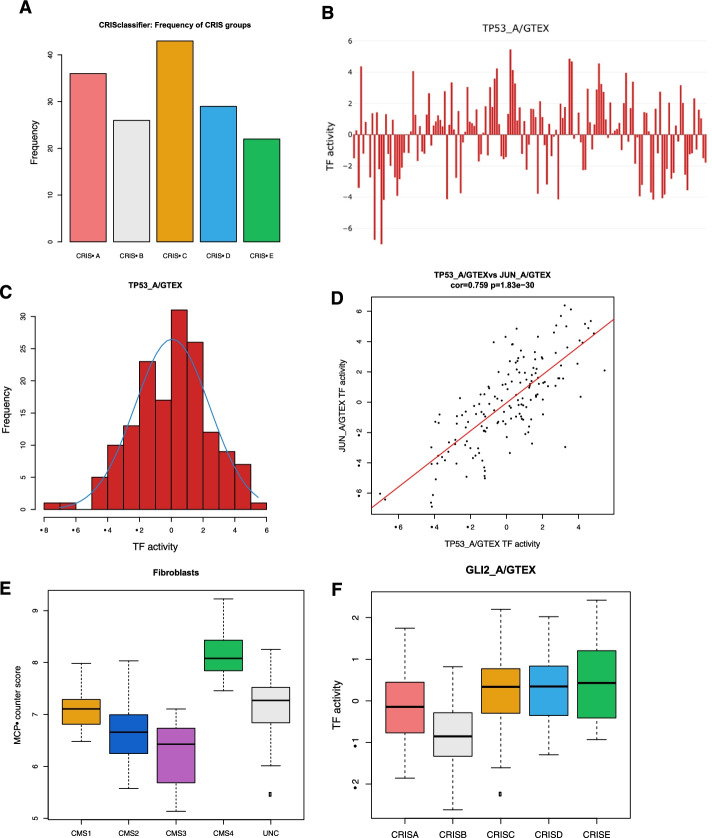
Characterisation of Stage II/III CRC (GSE103479) dataset in classifieR^c^. **a** Histogram depicting frequency of patients within each CRIS subgroup. **b** Interactive barplot showing TP53 transcription factor activity across all samples in the dataset. **c** Histogram showing the distribution of TP53 activity across all samples. **d** Dot plot visualising the correlation between TP53 and JUN transcription factor activity. **e** Box and whisker plot showing distribution of the MCP-counter score for Fibroblasts amongst the CMS subgroups. **f** Box and whisker plot showing distribution of the DoRothEA TF-activity score for GLI2 amongst the CRIS subgroups

When analysis of more than one tool is requested, classifieRc merges the outputs, such as CMS, CRIS, MCP-counter and DoRothEA scores based on sample ID into a single dowloadable table (.csv). This additionally enables interactive boxplot visualisation of the values generated by MCP-counter and DoRoTheA TF-activity and how these assessments segregate within the CRIS and CMS molecular subgroups as exemplified in Fig. 2. classifieRb in comparison merges the outputs of the genefu R package [16], with algorithms for PAM50 subtyping and these outputs are merged with MCP-counter and DoRothEA scores.

The application has been configured, hosted and launched on a hosted Virtual Machine and can be easily accessed using any operating system (http://generatr.qub.ac.uk/classifieRc and http://generatr.qub.ac.uk/classifieRb, which runs an example analyses on data from GSE103479 [4]. Updated versions with fixes and additional features will be automatically updated when developed. A demo dataset is available which allows users to navigate the applications prior to using it.

## Results

The classifieR^C^ application is comprised of 3 main sections or tabs: Introduction, Data Input, Data Output. Molecular classification of samples with the classifieR^c^ app is performed in one step on the data input tab. Firstly, the user transcriptional data is uploaded on the Data Input tab, the application automatically detects if the data needs normalized and which technology the data has been derived from. If all these details are correct, the user can click the “Classify!” button to run the analysis to completion. Many of the advanced options are hidden purposefully to provide the user with a extremely user friendly experience.

classifieR^c^ has been configured to utilise a range of input formats to enable input of data generated from a variety of technologies, including gene expression microarrays and a range of RNA-seq platforms such as BioSpyder’s TempO-Seq targeted sequencing platform (Additional file 1: Fig. 1A). Data are first uploaded in.csv or.txt format with sample names in columns and gene symbol/ID in rows. Next the technology/platform used to generate the data is automatically selected in order to allow the conversion of gene probes/IDs from certain platforms to HUGO Gene Symbol and ENTREZ Gene ID, both of which are required by the R packages utilized within this application. If a technology is not available, a custom lookup table can be uploaded by the user which matches probe IDs to gene symbol and ENTREZ ID. The user can optionally selects the packages they wish to run with optional pre-processing options, such as prefiltering using CPM cut-off for lowly expressed genes, changing the probability threshold for CMS and switching to CMScaller or the CMS Single Sample Prediction instead of the default CMS Random Forest model. All these additional advanced settings are hidden and not required, so that the app can be run without any user selected customisation. The analysis is initiated by clicking the “Classify!” button, which triggers the R scripts in the backend to run manually. Settings can be adjusted post-analysis, and the analysed data will update accordingly.

After the data are processed and classification is complete, the user navigates to the ‘Data table’ page. This page presents the processed expression table with the Gene Symbol in the row names. If the data are normalized, then the normalized data matrix can be downloaded and processed for further analysis, otherwise the data can be normalized within the application using standard DeSeq2 based normalization for gene counts [17]. This page also includes an interactive barchart that enables users to visualize expression of individual genes across the uploaded samples for a simple exploratory analysis of the dataset. If normalization has been selected, then an additional plot will show gene count plotted against CPM, providing a suggestion for which pre-filter cutoff should be selected.

Each individual classifier has a unique page with more detailed information on each classifier, including specific tables and plots that can be generated and downloaded. These include histograms showing patient frequency within the subgroups (Fig. 2a), interactive bar charts (Fig. 2b), dot plots showing correlation between two selected continuous values (Fig. 2c) and an interactive histogram showing distribution of a continuous value (Fig. 2d). The utility of each plot or tool within the app is explained as well as how it can be interpreted. These data are integrated into a single interactive report page which collates data from all classifiers into one place (Additional file 1: Fig. 1D). A single table is generated showing sample name or ID with each result collated from all classifiers in the application. Plots on the report page are interactive, enabling the user to visualize the relative amount of a specific cell type or the activity of a transcription factor in real-time against the subgroups using box and whisker plots (Fig. 2e, f). This example specifically shows the known increase in fibroblasts within the stromal-rich CMS4 subgroup. All the plots and tables can be downloaded, and post-analysis can be carried out if required.

Specific settings within the app can be adjusted if it is required. If the data needs to be pre-filtered, then the counts-per-million (CPM) cut-off can be adjusted; a detailed explanation on how to choose the optimal cutoff values for this step is available in the ‘Introduction’ page of the application. If the user is using cell line data, the more cell line-specific CMScaller [18] package can be used in place of CMSclassifier [19]. The app by default uses the random forest CMS prediction (predictCMS.RF) from CMSclassifier (v1.0); however, this can be changed to use the single sample prediction function (predictCMS.SSP) if there are a low number of samples. The posterior probability cut-off for assigning CMS subgroups can also be changed to be more strict or lenient on the CMS classification. DoRothEA can be adjusted to allow for alterative transcription factor regulons such as the alternative TCGA PanCancer Regulon within DoRothEA, or the option to upload a custom regulon in.RData format. To limit batch effects and classification error, the more samples that are uploaded the greater the reliability of the classifiers, specifically in the case of random forest CMS; if there is a low sample number (< 40), data can be appended to a reference dataset (Additional file 1: Fig. 1B). classifieR is a novel user-friendly approach and alternative to using R for these types of analysis. The app is open-source and can be further improved in the future such as new classification algoritithms and possibly extending to single cell analysis. The app can be additionally updated to add new features as they are required in the future. The shiny applications for these can be found at generatr.qub.ac.uk/classifieRb and generatr.qub.ac.uk/classifieRc.

Each processing step within the application has been carefully optimized to reduce computational processing power and memory usage, cutting down the length of time required to run these analyses when compared to running it outside of classifieR with 46% less memory usage overall and 78% faster to run a complete analysis of 156 samples.

## Conclusions

Currently, in order to perform the equivalent analyses, it requires an in-depth understanding of the R programming language and how to integrate multiple tools and outputs. classifieR enables labs without access to a dedicated bioinformation to understand what their data reveals functionally, how to interpret their results and to obtain information on the molecular makeup of their samples, providing an insight into patient prognosis and reponse to therapy. classifieR permits broader accessibility to tools that are currently only available through bioinformaticians and provides a faster and simultaneous analysis than through using the individual tools alone. Both applications are freely available to use at http://generatr.qub.ac.uk/

### Availability and requirements

- Project name: classifieR.
- Project home page: https://generatr.qub.ac.uk/
- Operating system(s): Platform Agnostic.
- Programming language: R version 4.0.3 (2020–10-10) / Java / HTML.
- Other requirements: Web browser.
- License: NA (Web server).

Any restrictions to use by non-academics: Research use only.

## Supplementary Information

**Additional file 1:** Supplementary figures. **Supplementary 1.** Screenshots of the classifieR application. (**A**) Screenshot of main page, showing where the file is selected, what technology is used and a submission button. (**B**) Progress bar showing progress of the app’s stratification. (**C**) An example of the advanced settings that can be selected when running the analysis (**D**) This page of the app Includes a table with all classification data, and box plots which integrate the classifiers (CRIS/CMS) with immune population and transcription factor activity. (**E**) This page has a detailed table and interactive heatmap (not shown), an interactive bar chart showing transcription factor activity, a histogram of activity across all samples and a correlation plot across all samples in which the transcription factor of interest can be selected. **Supplementary 2.** Sample number can confound transcriptional subtyping of GSE103479. (**A**) Schematic of experimental plan to test number of samples for robust CRIS/CMS classification (**B**) Sample discordance between subsets of samples against all samples ran simultaneously in GSE103479.

## Data Availability

The CRC dataset used in this article for demonstration was downloaded from the National Center for Biotechnology Information Gene Expression Omnibus repository with accession number GSE103479 [4]. The Breast Cancer dataset was downloaded and subsetted for breast cancer cell lines from Cancer Cell Line Encyclopedia [20].

## Abbreviations

CRC: Colorectal cancer
CRIS: Colorectal intrinsic subtypes
CMS: Consensus molecular subtypes
NGS: Next generation sequencing
MCP: Microenvironment cell population
GUI: Graphical user interface
TF: Transcription factor
PAM50: Prediction analysis of microarray 50
PDX: Patient-derived xenografts
SSP: Single sample prediction
RF: Random forest
TME: Tumour microenvironment
MCP: Multiple cell population
ssGSEA: Single sample gene set enrrichment analysis
