# Supplementary material for: classifieR a flexible interactive cloud-application for functional annotation of cancer transcriptomes
Source: BMC Bioinformatics. 2022 Mar 31;23:114. doi: 10.1186/s12859-022-04641-x (PMC8974006; doi:10.1186/s12859-022-04641-x)
Supplement: Supplementary file 1 — Additional file 1: Supplementary figures. Supplementary 1. Screenshots of the classifieR application. (A) Screenshot of main page, showing where the file is selected, what technology is used and a submission button. (B) Progress bar showing progress of the app’s stratification. (C) An example of the advanced settings that can be selected when running the analysis (D) This page of the app Includes a table with all classification data, and box plots which integrate the classifiers (CRIS/CMS) with immune population and transcription factor activity. (E) This page has a detailed table and interactive heatmap (not shown), an interactive bar chart showing transcription factor activity, a histogram of activity across all samples and a correlation plot across all samples in which the transcription factor of interest can be selected. Supplementary 2. Sample number can confound transcriptional subtyping of GSE103479. (A) Schematic of experimental plan to test number of samples for robust CRIS/CMS classification (B) Sample discordance between subsets of samples against all samples ran simultaneously in GSE103479. [file 12859_2022_4641_MOESM1_ESM.docx]

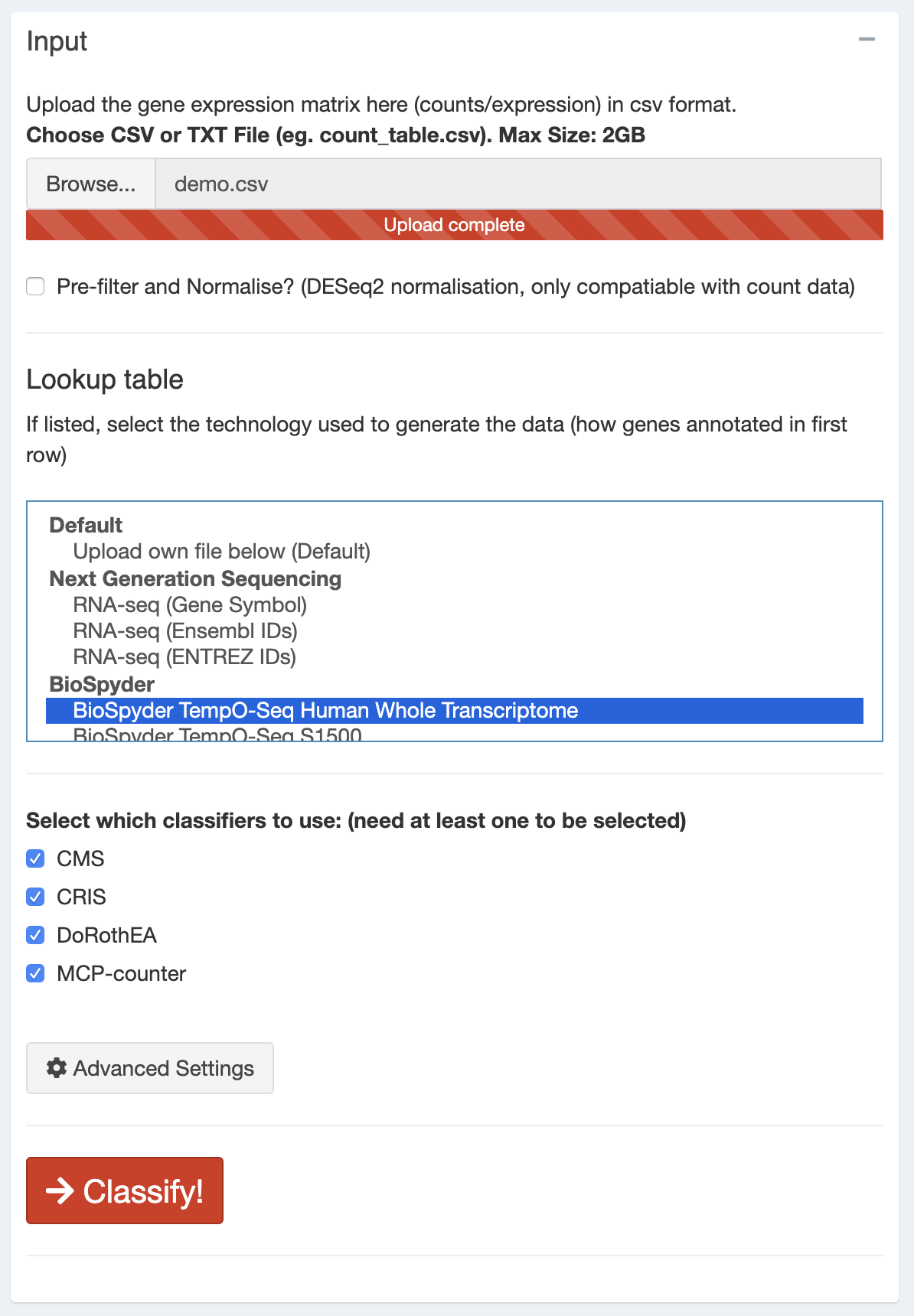

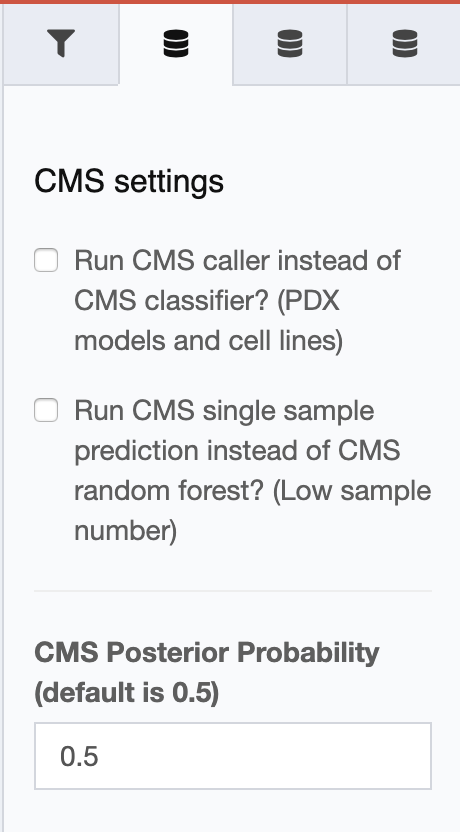

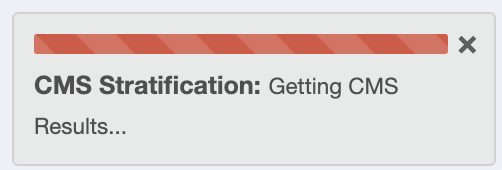

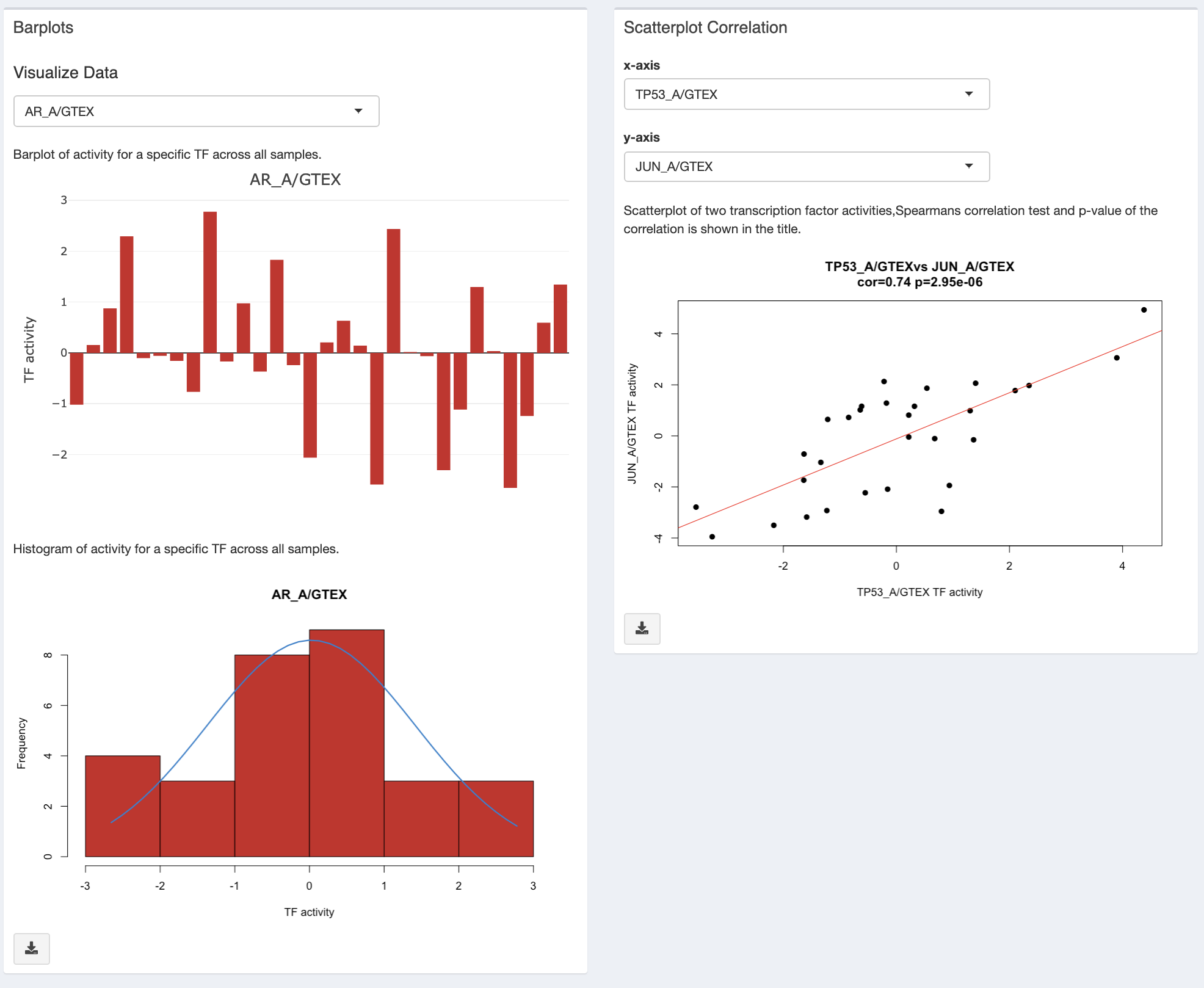

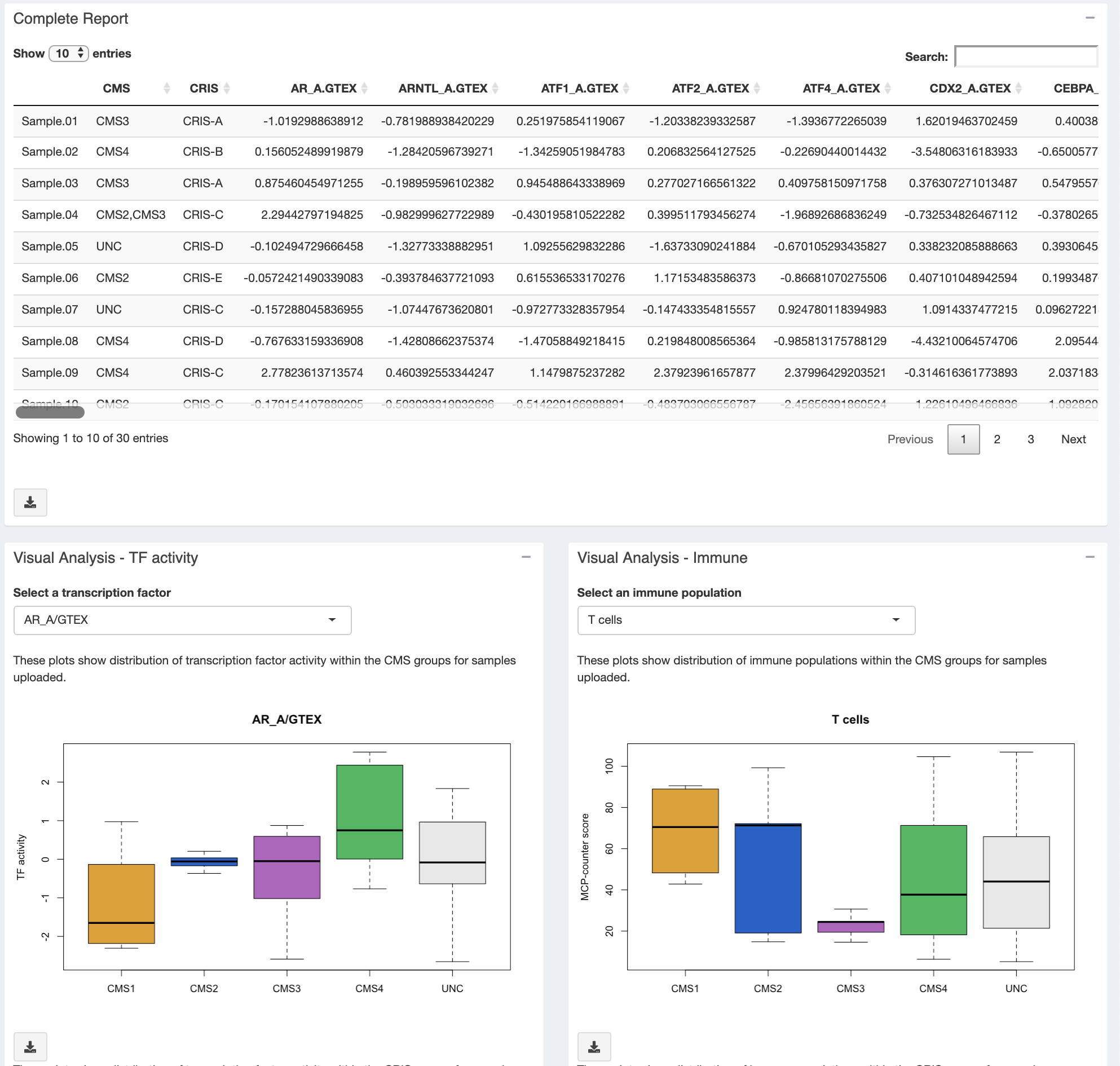


**Supplementary 1: Screenshots of the classifieR application. (A) Screenshot of main page, showing where the file is selected, what technology is used and a submission button. (B) Progress bar showing progress of the app’s stratification. (C) An example of the advanced settings that can be selected when running the analysis (D) This page of the app Includes a table with all classification data, and box plots which integrate the classifiers (CRIS/CMS) with immune population and transcription factor activity. (E) This page has a detailed table and interactive heatmap (not shown), an interactive bar chart showing transcription factor activity, a histogram of activity across all samples and a correlation plot across all samples in which the transcription factor of interest can be selected.**

**A**

**B**

**C**

**D**

**E**


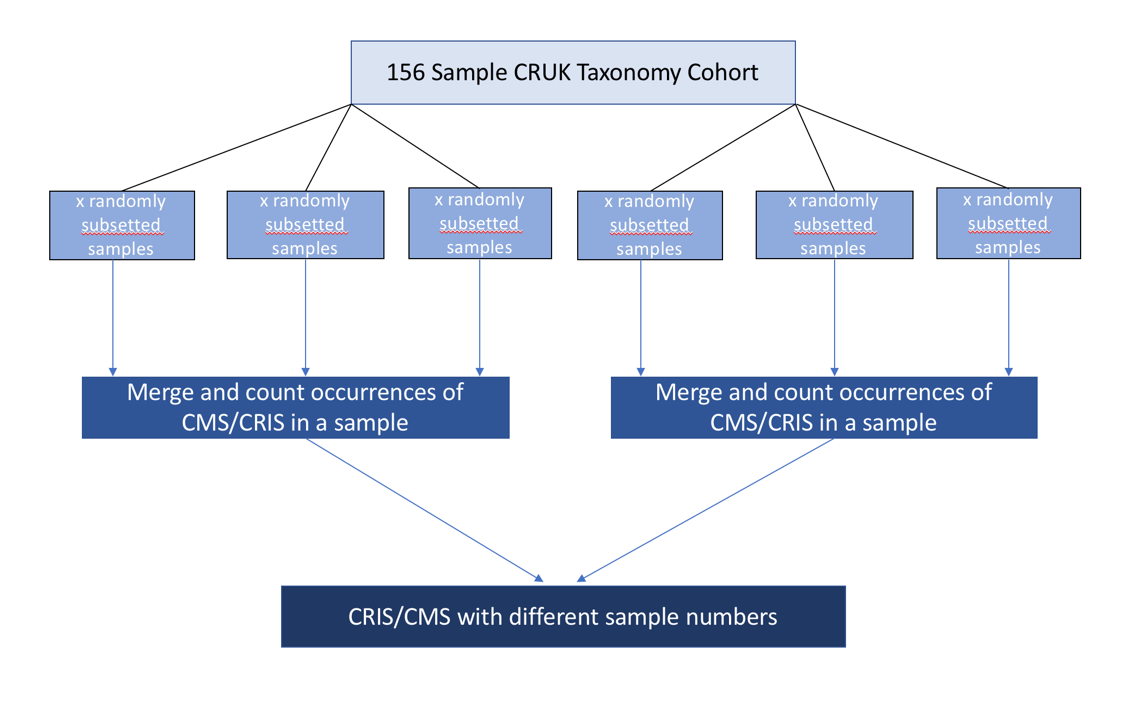

.RF

**Supplementary 2. Sample number can confound transcriptional subtyping of GSE103479. (A) Schematic of experimental plan to test number of samples for robust CRIS/CMS classification (B) Sample discordance between subsets of samples against all samples ran simultaneously in GSE103479.**

**B**

**A**
